# More than just statics: Static and temporal dynamic changes in intrinsic brain activity in unilateral temporal lobe epilepsy

**DOI:** 10.3389/fnhum.2022.971062

**Published:** 2022-08-31

**Authors:** Chengru Song, Xiaonan Zhang, Shaoqiang Han, Keran Ma, Kefan Wang, Xinyue Mao, Yajun Lian, Xianchang Zhang, Jinxia Zhu, Yong Zhang, Jingliang Cheng

**Affiliations:** ^1^Department of Magnetic Resonance Imaging, The First Affiliated Hospital of Zhengzhou University, Zhengzhou, China; ^2^Key Laboratory for Functional Magnetic Resonance Imaging and Molecular Imaging of Henan Province, Zhengzhou, China; ^3^Engineering Technology Research Center for Detection and Application of Brain Function of Henan Province, Zhengzhou, China; ^4^Engineering Research Center of Medical Imaging Intelligent Diagnosis and Treatment of Henan Province, Zhengzhou, China; ^5^Key Laboratory of Magnetic Resonance and Brain Function of Henan Province, Zhengzhou, China; ^6^Key Laboratory of Brain Function and Cognitive Magnetic Resonance Imaging of Zhengzhou, Zhengzhou, China; ^7^Key Laboratory of Imaging Intelligence Research Medicine of Henan Province, Zhengzhou, China; ^8^Department of Neurology, The First Affiliated Hospital of Zhengzhou University, Zhengzhou, China; ^9^MR Collaboration, Siemens Healthcare Ltd., Beijing, China

**Keywords:** cognition, dynamic, intrinsic brain activity, resting-state functional magnetic resonance imaging, temporal lobe epilepsy

## Abstract

**Background:**

Temporal lobe epilepsy (TLE) is the most prevalent refractory focal epilepsy and is more likely accompanied by cognitive impairment. The fully understanding of the neuronal activity underlying TLE is of great significance.

**Objective:**

This study aimed to comprehensively explore the potential brain activity abnormalities affected by TLE and detect whether the changes were associated with cognition.

**Methods:**

Six static intrinsic brain activity (IBA) indicators [amplitude of low-frequency fluctuation (ALFF), fractional ALFF (fALFF), regional homogeneity (ReHo), degree centrality (DC), global signal correlation (GSCorr), and voxel-mirrored homotopic connectivity (VMHC)] and their corresponding dynamic indicators, such as dynamic ALFF (dALFF), dynamic fALFF (dfALFF), dynamic ReHo (dReHo), dynamic DC (dDC), dynamic VMHC (dVMHC), and dynamic GSCorr (dGSCorr), in 57 patients with unilateral TLE and 42 healthy volunteers were compared. Correlation analyses were also performed between these indicators in areas displaying group differences and cognitive function, epilepsy duration, and severity.

**Results:**

Marked overlap was present among the abnormal brain regions detected using various static and dynamic indicators, primarily including increased ALFF/dALFF/fALFF in the bilateral medial temporal lobe and thalamus, decreased ALFF/dALFF/fALFF in the frontal lobe contralateral to the epileptogenic side, decreased fALFF, ReHo, dReHo, DC, dDC, GSCorr, dGSCorr, and VMHC in the temporal neocortex ipsilateral to the epileptogenic foci, decreased dReHo, dDC, dGSCorr, and dVMHC in the occipital lobe, and increased ALFF, fALFF, dfALFF, ReHo, and DC in the supplementary motor area ipsilateral to the epileptogenic foci. Furthermore, most IBA indicators in the abnormal brain region significantly correlated with the duration of epilepsy and several cognitive scale scores (*P* < 0.05).

**Conclusion:**

The combined application of static and dynamic IBA indicators could comprehensively reveal more real abnormal neuronal activity and the impairment and compensatory mechanisms of cognitive function in TLE. Moreover, it might help in the lateralization of epileptogenic foci and exploration of the transmission and inhibition pathways of epileptic activity.

## Introduction

As one of the most common cerebral diseases, epilepsy affects more than 70 million people worldwide (Thijs et al., 2019). Temporal lobe epilepsy (TLE) is the most prevalent refractory focal epilepsy (Engel, 2001), and is more likely accompanied by cognitive impairment, particularly in memory and language (Allone et al., 2017). It also impairs advanced social cognition, such as perception and personality (Schacher et al., 2006), accompanied by depression, anxiety, or other psychiatric symptoms (Keezer et al., 2016). Considering the high morbidity, severe cognitive problems, and poor quality of life, the neuropathologic mechanisms underlying TLE should be fully understood to facilitate the development of a more precise diagnosis and effective treatment (Wang et al., 2021).

Temporal lobe epilepsy is a disorder of large neural networks affecting brain activity (Spencer, 2002; Cataldi et al., 2013). Spencer (2002) first proposed the concept of “medial temporal/limbic network” in 2002, believing that it was bilateral, cortical, and subcortical, and included hippocampi, amygdala, entorhinal cortices, lateral temporal neocortices, and extratemporal components of the medial thalamus and inferior frontal lobes. In addition, Blumenfeld et al. (2004), (Norden and Blumenfeld, 2002; Blumenfeld and Taylor, 2003) proposed a “network inhibition hypothesis” in which complex partial seizures arising in the temporal lobe propagated to the medial thalamus and upper brainstem reticular formation, disrupting their normal activating functions. This, in turn, might lead secondarily to functional inactivation of widespread regions of the frontal and parietal association cortices, causing the loss of consciousness. Thus, anatomical structures that are abnormally activated or inhibited could contribute to epileptic symptoms, impaired cognitive function (Holmes et al., 2014; Burianová et al., 2017), or psychiatric complications (Jiang et al., 2018). The existence and importance of the temporal epilepsy network have been confirmed using various modalities (Spencer, 2002), such as clinical observations, EEG (Bettus et al., 2008), SPECT (Blumenfeld et al., 2004), PET (Concha et al., 2005), volumetric (Bernasconi et al., 2003; Araújo et al., 2006), and functional MRI (Zhang et al., 2010; Maneshi et al., 2014). The study of the TLE network might contribute to our in-depth understanding of the TLE pathophysiologic mechanism and is expected to be an auxiliary tool for the clinical diagnosis or provide a new direction for the treatment.

Blood oxygenation level-dependent (BOLD) resting-state functional magnetic resonance imaging (rs-fMRI) focuses on spontaneous/intrinsic fluctuations of BOLD signals in a low-frequency band (<0.1 Hz). Its functional significance was first proposed by Biswal et al. (1995). Intrinsic brain activity (IBA) plays a vital role in brain function (Fox and Raichle, 2007; Raichle, 2010, 2015). A growing number of rs-fMRI-based IBA measurements have been proposed, such as functional connectivity (FC) (Biswal et al., 1995), amplitude of low-frequency fluctuation (ALFF) (Zang et al., 2007), fractional ALFF (fALFF) (Zou et al., 2008), regional homogeneity (ReHo) (Zang et al., 2004), degree centrality (DC) (Zuo et al., 2012), voxel-mirrored homotopic connectivity (VMHC) (Zuo et al., 2010), global signal correlation (GSCorr) (Yan et al., 2017), and independent component analysis (ICA) (Beckmann et al., 2005). Each method aims to reveal a distinct aspect of intrinsic brain function (Margulies et al., 2010). The distinctions and commonalities among these measures are yet to be fully explored (Yan et al., 2017).

Temporal lobe epilepsy can affect brain activity. Zhang et al. (2010) and Singh et al. (2020) demonstrated that patients with TLE had increasing ALFF in the mesial temporal epilepsy network regions and decreasing ALFF in the default-mode network (DMN) regions. Zhang et al. (2010) also demonstrated that the increases in ALFF and ReHo might be associated with the interictal epileptiform discharges, and ALFF might be applied for the lateralization. Previous studies have focused mainly on ALFF, fALFF, or ReHo (Zeng et al., 2013; Zhao et al., 2020), and the results were partially inconsistent. Studies on VMHC (Xu et al., 2014; Shi et al., 2021), DC (Liang et al., 2020; Nakai et al., 2021), and GSCorr are rare or even missing.

Moreover, the aforementioned traditional IBA measurements are static, assuming that the BOLD signal is stationary during the scanning period (Liao et al., 2014). However, the activity of the human brain is environmentally sensitive and activity-dependent, implying that the IBA is highly dynamic and fluctuates over time, which underlies the integration of brain function (Park et al., 2018). Thus, static IBA metrics are insufficient to describe the transient changes in IBA. Fortunately, recent investigations of brain activity have taken fluctuation over time into account, which can be quantified by measuring the temporal variance through the sliding window approach (Chen et al., 2017). By calculating the standard deviation (SD) or variability of the aforementioned metrics in all the windows, we can acquire dynamic IBA metrics, such as dynamic FC (dFC), dynamic ALFF (dALFF), dynamic fALFF (dfALFF), dynamic ReHo (dReHo), dynamic DC (dDC), dynamic VMHC (dVMHC), and dynamic GSCorr (dGSCorr). These metrics, extended from static to dynamic, reflect the dynamic change degree of each index in the time dimension. They not only further reflect rich dynamic information of IBA, but also proved to have high stability (Yan et al., 2017).

In a literature search of the aforementioned IBA dynamic indicators, most research on TLE was found to focus on dynamic FC studies (Jiang et al., 2020; Li et al., 2022; Pang et al., 2022). However, the relevant studies which applied the other dynamic indicators, such as dALFF, dfALFF, dReHo, dDC, dVMHC and dGSCorr, in TLE patients could not be retrieved. Only one piece of literature has been retrieved to investigate the dALFF alteration in benign childhood epilepsy (BECTS) (Morgan et al., 2020).

Consequently, this study was performed to comprehensively understand the abnormalities of IBA in patients with TLE and analyze the consistency and complementarity of various indicators. We studied the static and temporal dynamic characteristics of IBA in patients with unilateral TLE from the perspectives of local activity (ALFF and fALFF), local synchronization or connectivity (ReHo and VMHC), and the whole brain connectivity (DC and GSCorr). Moreover, the correlations between abnormal IBA and cognition, epilepsy duration, or severity were explored. These might enhance the understanding of the relationship between TLE and cognition deficits.

## Materials and methods

### Participants

The participants were divided into two groups. The TLE group was comprised of 64 patients with unilateral TLE who had consulted the Neurology Clinic and Inpatient Department of The First Affiliated Hospital of Zhengzhou University. The healthy control (HC) group was comprised of 45 healthy volunteers from the local communities matched for age and sex.

The diagnosis was made based on a detailed history, neurologic examination, electroencephalography (EEG) recordings, and imaging findings. The inclusion criteria for patients were as follows: (1) all right-handed, (2) >14 years old, and (3) met any 2 or more of the following criteria: ① clinical semiology consistent with seizures of the unilateral left/right temporal lobe origin, ② interictal or ictal epileptic discharges of the unilateral left/right temporal lobe origin according to scalp EEG or intracranial electrode EEG, ③ MRI results that suggested the left/right hippocampal sclerosis of the patients or positron emission tomography (PET) results that suggested the left/right hypometabolic temporal lobe. The exclusion criteria for patients were as follows: (1) patients with brain structural abnormalities (except for hippocampal sclerosis), other systemic diseases, psychiatric disorders, history of alcohol abuse, and so forth, (2) unidentified lateralization of TLE, and (3) patients who have taken antiepileptic drugs regularly.

The inclusion criteria for controls were as follows: (1) normal head MRI results, (2) >14 years old, and (3) all right-handed. The exclusion criteria for controls were as follows: patients with any prior history of neurologic or psychiatric diseases, history of alcohol or cigarette abuse, and so forth. Handedness was rated according to the Edinburgh Handedness Inventory.

This study was approved by the Research Ethics Committee at The First Affiliated Hospital of Zhengzhou University (No. 2019-KY-232), and informed consent was obtained from all participants or their legal guardian/next of kin.

### MRI data acquisition

Resting-state fMRI data were acquired using a 3-T Magnetom Prisma MRI scanner (Siemens Healthcare, Erlangen, Germany) with a 64-channel head coil. All participants were advised to lie down flat, not think, close their eyes, and breathe quietly. 3D T1-weighted structural images were acquired using a magnetization prepared rapid acquisition gradient echo sequence with the following parameters: TR/TE = 2,300/2.32 ms; FOV = 240 mm × 240 mm; slice number = 176; and voxel size = 0.9 × 0.9 × 0.9 mm^3^. Functional images were acquired using a simultaneous multi-slice (SMS) echo-planar imaging sequence with the following parameters and a total scan time of 400 s: TR/TE = 1,000/30 ms; FOV = 220 × 220 mm^2^; slice thickness = 2.2 mm; flip angle = 70; voxel size = 2.0 × 2.0 × 2.2 mm^3^; slice number = 52; volumes = 400; SMS = 4.

### Clinical data and neuropsychologic tests

For all the participants in the TLE and HC groups, the clinical data such as sex, age, education years, and epilepsy duration years were recorded. All participants underwent national hospital seizure severity scale (NHS3) testing and neuropsychologic testing prior to scanning to measure their seizure severity and cognitive function. The neuropsychologic test included Mini-Mental State Examination (MMSE), memory and executive screening (MES), Montreal cognitive assessment-basic (MOCA-B), auditory verbal learning test (AVLT), and shape trail making test-A/B (STT-A/B). The AVLT test included 6 scores: immediate recall scores (N1, N2, and N3), 5-min short delayed recall score (N4), 20-min long-delayed recall score (N5), and reconfiguration. The scales used in this study were all in Mandarin Chinese versions. The aforementioned scale was evaluated by the same physician who had passed the systematic training and examination.

### Data analysis

#### rs-fMRI preprocessing

The rs-fMRI data were preprocessed using the Data Processing Assistant for Resting-State fMRI Analysis Toolkit (DPARSF, V5.2) (Chao-Gan and Yu-Feng, 2010). The first 10 volumes were deleted, and then slice-timing and realignment were performed. Participants with head motion of >2.5 mm in maximum displacement or >2.5 rotation in angular motion were excluded. Seven patients with TLE and 3 healthy controls were excluded because of excessive head motion. Next, the functional images were spatially normalized to the standard EPI template and resampled to 3 × 3 × 3 mm^3^. Several spurious variances, including 24 head motion parameters, cerebrospinal fluid signals, and white matter signals, were regressed by multiple linear regression analysis. Moreover, the time points whose mean frame-wise displacement (FD) (Power et al., 2012) value exceeded 0.2 mm and the linear regression factor were also regressed in this step. Subsequently, functional images were temporal bandpass filtered between 0.01 and 0.1 Hz.

#### Calculation of static amplitude of low-frequency fluctuation, fractional amplitude of low-frequency fluctuation, regional homogeneity, degree centrality, global signal correlation, and voxel-mirrored homotopic connectivity

The Data Processing and Analysis of Brain Imaging (DPABI, V6.0_210501) toolbox (Yan et al., 2016) was used to calculate ALFF, fALFF, ReHo, DC, GSCorr, and VMHC. The calculation of ALFF was based on the low-frequency range from 0.01 to 0.1 Hz. Then, we calculated fALFF by obtaining the ratio of the power spectrum of low frequency (0.01–0.1 Hz) to that of the entire frequency range (0–0.25 Hz). For ReHo, Kendall’s coefficient of concordance of the time course of every 27 nearest neighboring voxels was calculated (Zang et al., 2004). DC (Zuo et al., 2012) was defined as the weighted sum of voxels whose correlation coefficient with all the other voxels in the whole brain was above the threshold (*r* > 0.25 in this study). GSCorr (Power et al., 2017) was calculated as the Pearson correlation between the global signal and all the other voxels in the whole brain with Fisher Z-transformation. Before calculating VMHC, T1 image segmentation and smooth (with a 6-mm full width at half-maximum (FWHM)] Gaussian kernel were processed. Considering the differences in geometric properties between the bilateral cerebral hemispheres, we re-normalized each T1 structure image to the Montreal Neurological Institute (MNI) template with symmetrical left and right brains using non-linear registration. The foregoing MNI templates were applied to the processed fMRI data. The functional homotopy of each pair of mirrored voxels was calculated using Pearson’s correlation coefficient. Subsequently, the Fisher Z-transformation was used to convert the coefficient values into VMHC values.

#### Calculation of dynamic amplitude of low-frequency fluctuation, fractional amplitude of low-frequency fluctuation, regional homogeneity, degree centrality, global signal correlation, and voxel-mirrored homotopic connectivity

The dynamic parameters (dALFF, dfALFF, dReHo, dDC, dGSCorr, and VMHC) were analyzed using temporal dynamic analysis toolkits (Yan et al., 2017) based on DPABI. An approach based on a sliding window, which was sensitive in detecting time-dependent variations, was performed to characterize the temporal dynamic patterns (Hindriks et al., 2016). Ideally, the length of a window should be small enough to monitor potentially transient signals and large enough to analyze the lowest fluctuations of interest in the signals (Sakoğlu et al., 2010). Previous studies on sliding window connectivity have applied a sliding window length as small as 10 s (Thompson et al., 2013) and as long as 180 s (Gonzalez-Castillo et al., 2015). In this study, a moderate-length sliding rectwin window of 60 TRs (60 s) and a shifting step size of 30 TRs (30 s) were applied. We also examined the effect with other window lengths and step sizes, and they were included in validation analyses.

To track the dALFF (similar to other indicators), we used a sliding-window approach to separate the full-length BOLD fMRI time series into a series of small data segments (60 TRs in this study). After applying the rectwin windows to BOLD signals, we obtained a windowed time series of 12 windows in total. Then, within each window, we calculated the ALFF for every voxel in the brain. The sliding window was systematically shifted by 30 TRs and the corresponding ALFF was computed. This process was performed until the entire data length was covered. The temporal variability of dALFF was defined as the standard deviation (SD) of dALFF maps across time windows.

The maps of all the static and dynamic indicators were Z-standardized (Yan et al., 2016) within a gray matter mask across all the voxels and then spatially smoothed (except VMHC) with a Gaussian kernel of 6-mm FWHM to reduce the global effects of variability across participants and improve the signal-to-noise ratio.

For expanding the study sample size and explaining the group changes as either ipsilateral or contralateral to left temporal lobe epilepsy (LTLE) (Blumenfeld et al., 2009; Zeng et al., 2013), we right/left flipped the maps of the 27 patients with right temporal lobe epilepsy (RTLE) to obtain the mirror copies as LTLE and then finally obtained the data of 57 LTLE for the subsequent statistical analysis.

### Statistical analysis

The demographic and cognitive scale scores were evaluated between the groups of TLE and HC using SPSS software (version 17.0; IBM, IL, United States). Differences in sex were analyzed with a chi-square test (*P* < 0.05). Differences in age and cognitive scale scores were analyzed with a 2-sample *t*-test or a non-parametric test (*P* < 0.05).

To examine the between-group differences in the 12 measurements of static and dynamic indicators, we employed a 2-sample *t*-test using DPABI, with age, sex, education, and mean FD as covariates. Multiple comparison correction was performed based on the Gaussian random field theory (GRF, voxelwise *P* < 0.005, cluster-wise *P* < 0.05). A similar group comparison was also applied between LTLE and RTLE groups. The figures were drawn using both DPABI and BrainNet Viewer (Xia et al., 2013).

### Correlation analysis

With the peak voxels of abnormal regions as spherical centers, spherical regions of intrest (ROIs) were constructed around these abnormal regions (with a 6-mm radius). We used *Spearman* correlation analysis with SPSS software (version 17.0; IBM, IL, United States) to assess the relationship between metrics in these abnormal regions and the epilepsy duration, NHS3, and cognitive scores. Statistical significance was defined as *P* < 0.05.

### Validation analyses

We carried out additional analyses to validate our findings of the dynamic IBA indicators. We examined the difference with other window sizes/step sizes (60/10 TRs and 80/10 TRs) to validate our results.

## Results

### Clinical information and neuropsychologic results

The demographic, clinical, and neuropsychologic characteristics of 57 patients with TLE and 42 HCs are listed in Table 1. No remarkable differences were noted in age and sex between the TLE and HC groups (*P* > 0.05). Cognitive decline was observed in patients with TLE. MMSE, MES, MOCA-B, and AVLT (N1, N2, N3, N4, N5, and recognition) all decreased in the TLE group compared with the HC group (*P* < 0.05). However, STT-A and STT-B increased in the TLE group (*P* < 0.05).

**TABLE 1:** Demographic, clinical, and neuropsychological data.

	TLE	HC	Statistic	*P*-value
Age (year)	28.77 ± 11.26	26.79 ± 9.02	Z = –0.716	0.474
Sex (M/F)	25/32	19/23	χ^2^ = 0.019	0.891
Epilepsy duration (y)	6.91 ± 8.04	–	–	–
NHS3	8.82 ± 3.33	–	–	–
MMSE	28.25 ± 1.86	29.10 ± 0.96	Z = –2.206	0.027*
MES	84.35 ± 13.32	95.43 ± 4.51	Z = –5.339	<0.001*
MOCA-B	24.47 ± 3.56	27.45 ± 2.07	Z = –4.572	<0.001*
N1	3.98 ± 1.66	5.36 ± 1.59	Z = –3.800	<0.001*
N2	6.21 ± 1.87	7.17 ± 2.17	Z = –2.342	0.019*
N3	7.54 ± 2.36	8.67 ± 2.00	Z = –2.501	0.012*
N4	5.74 ± 2.95	7.86 ± 1.93	Z = –3.775	<0.001*
N5	4.74 ± 3.00	6.88 ± 2.52	Z = –3.542	<0.001*
Reconfiguration	20.44 ± 2.82	22.40 ± 2.51	Z = –4.266	<0.001*
STT-A (s)	51.91 ± 22.58	38.34 ± 12.34	Z = –3.289	0.001*
STT-B (s)	125.39 ± 54.41	88.30 ± 24.46	Z = –4.559	<0.001*

Data represent mean ± standard deviation; *indicates that the difference in data between the two groups was statistically significant. HC, healthy control; MES, memory and executive screening; MMSE, Mini-Mental State Examination; MOCA-B, Montreal cognitive assessment-basic; NHS3, national hospital seizure severity scale; STT-A, shape trail making test-A; STT-B, shape trail making test-B; TLE, temporal lobe epilepsy.

### Group differences of intrinsic brain activity indicators between temporal lobe epilepsy and healthy control

#### Intergroup differences in amplitude of low-frequency fluctuation and dynamic amplitude of low-frequency fluctuation

**FIGURE 1:**
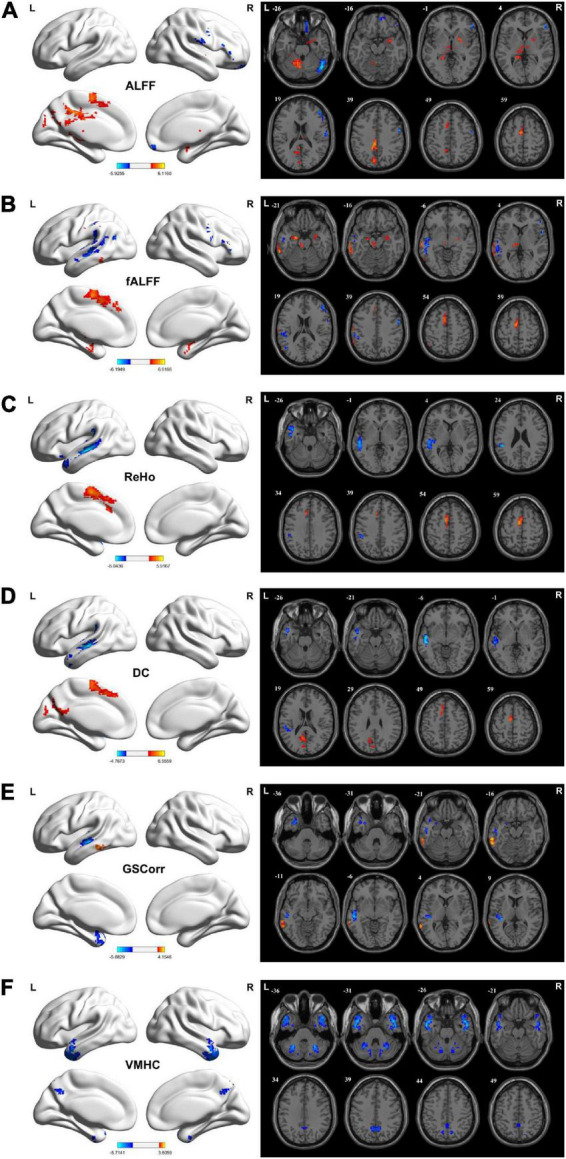
Brain regions showing considerably altered static ALFF **(A)**, fALFF **(B)**, ReHo **(C)**, DC **(D)**, GSCorr **(E)**, and VMHC **(F)** between TLE and HCs groups. GRF corrected; voxel-wise *P* < 0.005, cluster-wise *P* < 0.05. The color bar indicates the *t*-value. Warm colors indicated increased indicator values, while cold colors indicated decreased indicator values. ALFF, amplitude of low-frequency fluctuation; DC, degree centrality; fALFF, fractional ALFF; GRF, Gaussian random field theory; GSCorr, global signal correlation; ReHo, regional homogeneity; TLE, temporal lobe epilepsy; VMHC, voxel-mirrored homotopic connectivity.

Increased ALFF values were observed in the left median cingulate gyrus (MCG), posterior cingulate gyrus (PCG), cerebellum 4_5/6/8/9, hippocampus, thalamus, supplementary motor area (SMA), precuneus, cuneus, right pallidum, lenticula, amygdala, and parahippocampal gyrus (PHG) in the TLE group compared with the HC group. However, TLE displayed decreasing ALFF values in the right cerebellum Crus1, precentral gyrus (PreCG), postcentral gyrus (PostCG), triangular inferior frontal gyrus (TIFG), middle frontal gyrus (MFG), and orbital frontal gyrus (OFG) (GRF corrected, *P*_voxel_ < 0.005, *P*_*cluster*_ < 0.05; Figure 1A and Table 2).

**TABLE 2:** Brain regions with significant differences of IBA indicators between TLE and HC groups.

Measurements	Brain regions	MNI coordinates			Cluster size (Voxel)	Peak *t*-value
		
		x	y	z		
ALFF	MCG_L, PCG_L, and Precuneus_L	–6	–33	39	171	5.52
	Cerebellum 4_5/6/8/9_L	–12	–57	–21	149	4.36
	Hippocampus_L and Thalamus_L	–21	–42	0	113	3.71
	SMA_L	–6	–6	60	110	4.56
	Precuneus_L and Cuneus_L	–9	–75	39	95	3.84
	Pallidum_R, lenticula_R, Amygdala_R, and PHG_R	18	3	–3	135	4.01
	Cerebellum_Crus1_R	48	–66	–24	159	–5.93
	PreCG_R and PostCG_R	60	–3	33	74	–4.73
	TIFG_R and MFG_R	51	36	0	71	–4.39
	OFG_R	18	57	–18	68	–3.99
dALFF	MCG_L/R, PCG_L, and Precuneus_L	-9	–36	39	248	4.96
	Cerebellum 4_5/6/8/10_L	–6	–60	–21	226	4.56
	Thalamus_L, Hippocampus_L, and Precuneus_L	–6	–33	9	186	4.30
	Thalamus_R, Hippocampus_R, and Basal ganglia_R, Amygdala_R and olfactory cortex_L/R	15	–12	3	492	5.46
	Cerebellum_Crus1_R	45	–66	–24	221	–5.79
	OFG_R, SFG_R, and Rectus gyrus_R	6	66	–3	183	–3.70
fALFF	Hippocampus_R, TP_R, and Amygdala_R	30	–5	118	81	4.80
	ITG_L, MTG_L, and SMG_L	-63	–42	–21	200	5.80
	SMA_L and SFG_L	–9	–6	57	150	5.47
	Thalamus_L	–15	–18	6	71	4.54
	Hippocampus_L and PHG_L	–21	–6	–21	60	5.07
	SMG_L, MTG_L, STG_L, and AG_L	–48	–36	24	329	–4.90
	PostCG_R, PreCG_R, MFG_R, and IFG_R	60	-3	33	129	–5.67
dfALFF	SMA_L and MSFG_L	–9	–3	57	144	4.65
	DSFG_R and SMA_R	21	24	51	128	5.61
ReHo	MTG_L, STG_L, ITG_L, TP_L, and SMG_L	–51	–24	-6	549	–4.89
	SMA_L and MSFG_L	–9	–6	57	170	4.83
dReHo	STG_L, Heschl’s gyrus_L, and SMG_L	–33	–33	12	102	–5.11
	MTG_L	–54	–39	-6	89	–4.19
	CAL_R and Cuneus_R	12	–90	12	97	–4.72
DC	MTG_L, STG_L, ITG_L, SMG_L, and Heschl’s gyrus_L	–51	–15	–6	317	–4.77
	Precuneus_L, Cuneus_L, and CAL_L	–9	–57	21	156	3.83
	SMA_L	–9	–6	57	134	4.42
dDC	CAL_L, SOG_L, Cuneus_L, and LG_L	–9	–102	–3	135	–6.99
	CAL_R and Cuneus_R	9	–87	15	72	–4.02
	CAL_R and Precuneus_R	21	–54	15	70	–6.46
	STG_L	–36	–36	15	65	–5.86
	MTG_L	–66	–51	–12	73	5.31
GSCorr	TP_L, MTG_L, STG_L, and ITG_L	–30	6	–36	107	–4.20
	MTG_L, STG_L, and Heschl’s gyrus_L	–36	–33	12	202	–5.36
	ITG_L and MTG_L	–60	–39	–18	127	4.04
dGSCorr	CAL_L, MOG_L, SOG_L, IOG_L, and Cuneus_L	–3	–102	–3	144	–6.87
	CAL_R, LG_R, and Cuneus_R	15	–99	0	117	–4.32
	STG_L and Heschl’s gyrus_L	–39	–36	15	129	–5.95
VMHC	Bilateral MTG, ITG, STG, and TP	± 51	–3	–27	452	–5.71
	Bilateral Cerebellum Crus1/Crus2/6	± 27	–57	–36	148	–4.77
	Bilateral Precuneus	0	–57	36	83	–3.64
dVMHC	Bilateral CAL and Cuneus	± 9	–96	18	130	–4.41
	Bilateral PCL	± 6	–33	72	61	–4.07

AG, angular gyrus; ALFF, amplitude of low-frequency fluctuation; CAL, calcarine cortex; dALFF, dynamic fractional ALFF; DC, degree centrality; dDC, dynamic degree centrality; dGSCorr; dynamic global signal correlation; dfALFF, dynamic fractional ALFF; dReHo, dynamic regional homogeneity; DSFG, dorsolateral superior frontal gyrus; fALFF, fractional ALFF; GSCorr, dynamic global signal correlation; HC, healthy control; IOG, inferior occipital gyrus; IBA, intrinsic brain activity; ITG, inferior temporal gyrus; L, left; LG, lingual gyrus; MCG, median cingulate gyrus; MFG, middle frontal gyrus; MOG, middle occipital gyrus; MSFG, medial superior frontal gyrus; MTG, middle temporal gyrus; OFG, orbital orbital frontal gyrus; PCG, posterior cingulate gyrus; PCL, paracentral lobule; PHG, parahippocampal gyrus; PostCG, poscentral gyrus; PreCG, precentral gyrus; R, right; ReHo, regional homogeneity; SFG, superior frontal gyrus; SMA, supplementary motor area; SMG, superior marginal gyrus; SOG, superior occipital gyrus; STG, superior temporal gyrus; TIFG, triangular inferior frontal gyrus; TLE; temporal lobe epilepsy; TP, temporal pole; VMHC, voxel-mirrored homotopic connectivity; dVMHC, dynamic voxel-mirrored homotopic connectivity.

**FIGURE 2:**
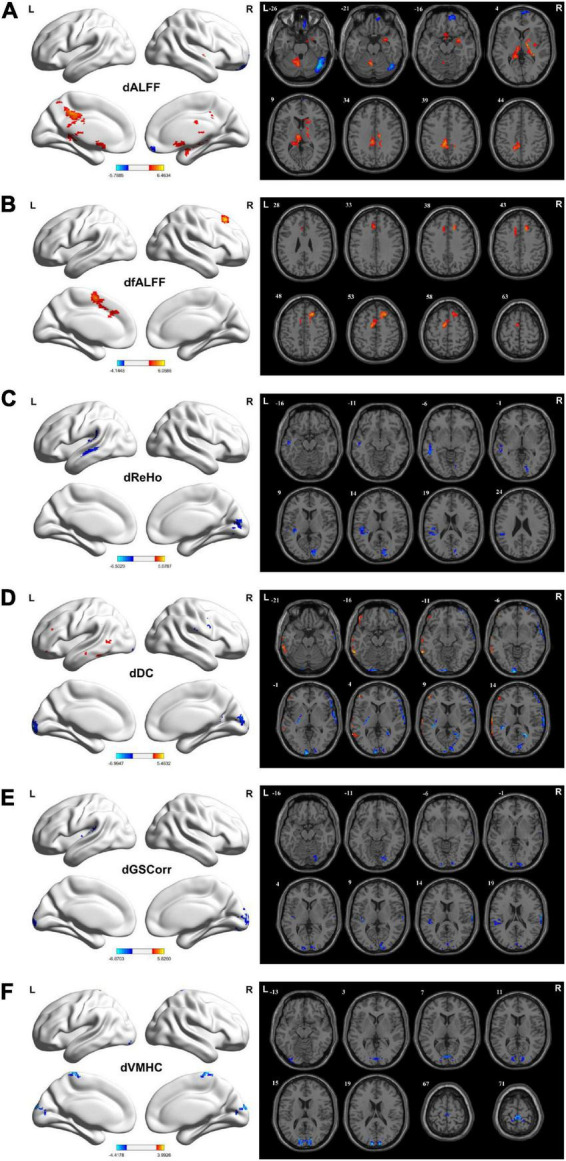
Brain regions showing considerably altered dALFF **(A)**, dfALFF **(B)**, dReHo **(C)**, dDC **(D)**, dGSCorr **(E)**, and dVMHC **(F)** between patients with TLE and HCs. GRF corrected; voxel-wise *P* < 0.005, cluster-wise *P* < 0.05. The color bar indicates the *t*-value. Warm colors indicated increased indicator values, while cold colors indicated decreased indicator values. dALFF, dynamic amplitude of low-frequency fluctuation; dfALFF, dynamic fractional ALFF; dDC, dynamic degree centrality; dGSCorr, dynamic global signal correlation; GRF, Gaussian random field theory; HCs, healthy controls; dReHo, dynamic regional homogeneity; TLE, temporal lobe epilepsy; dVMHC, dynamic voxel-mirrored homotopic connectivity.

Increased dALFF variability was observed in the bilateral MCG, left PCG, left precuneus, left cerebellum 4_5/6/8/10, bilateral hippocampus, thalamus, right basal ganglia, right amygdala, and bilateral olfactory cortex in the TLE group compared with the HC group. However, TLE displayed decreasing dALFF variability in the right OFG, superior frontal gyrus (SFG), and rectus gyrus (GRF corrected, *P*_voxel_ < 0.005, *P*_*cluster*_ < 0.05; Figure 2A and Table 2).

#### Intergroup differences in fractional amplitude of low-frequency fluctuation and dynamic amplitude of low-frequency fluctuation

Increased fALFF values were detected in the bilateral hippocampus, right amygdala, right temporal pole (TP), left middle temporal gyrus (MTG), inferior temporal gyrus (ITG), superior marginal gyrus (SMG), SMA, SFG, PHG, and thalamus in the TLE group compared with the HC group. However, the fALFF values decreased in the left SMG, MTG, superior temporal gyrus (STG), angular gyrus (AG), and right PreCG, PostCG, MFG, and IFG (GRF corrected, *P*_voxel_ < 0.005, *P*_*cluster*_ < 0.05; Figure 1B and Table 2).

Increased dfALFF variability was detected in the bilateral SMA, left medial superior frontal gyrus (MSFG), and right dorsolateral superior frontal gyrus (DSFG) in the TLE group compared with the HC group (GRF corrected, *P*_voxel_ < 0.005, *P*_*cluster*_ < 0.05; Figure 2B and Table 2).

#### Intergroup differences in regional homogeneity and dynamic regional homogeneity

Increased ReHo values in the left SMA and MSFG but decreased ReHo values in the left MTG, STG, ITG, TP, and SMG were detected in the TLE group compared with the HC group increased ReHo values were found in the left SMA, MSFG, but decreased ReHo values were found in the left MTG, STG, ITG, TP, SMG (GRF corrected, *P*_voxel_ < 0.005, *P*_*cluster*_ < 0.05; Figure 1C and Table 2).

For dReHo, TLE decreased in left STG, Heschl’s gyrus, SMG, MTG, and right calcarine cortex (CAL), and cuneus (GRF corrected, *P*_voxel_ < 0.005, *P*_*cluster*_ < 0.05; Figure 2C and Table 2).

#### Intergroup differences in degree centrality and dynamic degree centrality

Increased DC values in the left precuneus, cuneus, CAL, and SMA but decreased DC values in the left MTG, STG, ITG, SMG, and Heschl’s gyrus were noted in the TLE group compared with HC group (GRF corrected, *P*_voxel_ < 0.005, *P*_*cluster*_ < 0.05; Figure 1D and Table 2).

Increased dDC variability in the left MTG but decreased dDC variability in bilateral CAL and cuneus, left superior occipital gyrus (SOG), left lingual gyrus (LG), left STG, and right precuneus were detected in the TLE group compared with the HC group (GRF corrected, *P*_voxel_ < 0.005, *P*_*cluster*_ < 0.05; Figure 2D and Table 2).

#### Intergroup differences in global signal correlation and dynamic global signal correlation

Increased GSCorr values in the left MTG and ITG but decreased GSCorr values in the left TP, MTG, STG, ITG, and Heschl’s gyrus were observed in the TLE group compared with the HC group (GRF corrected, *P*_voxel_ < 0.005, *P*_*cluster*_ < 0.05; Figure 1E and Table 2).

Decreased dGSCorr variability was detected in the bilateral CAL, cuneus, left SOG, left middle occipital gyrus (MOG), left inferior occipital gyrus (IOG), right LG, left STG, and Heschl’s gyrus in the TLE group compared with the HC group (GRF corrected, *P*_voxel_ < 0.005, *P*_*cluster*_ < 0.05; Figure 2E and Table 2).

#### Intergroup differences in voxel-mirrored homotopic connectivity and dynamic voxel-mirrored homotopic connectivity

Decreased VMHC values were noted in the bilateral MTG, ITG, STG, TP, cerebellum Crus1/Crus2/6, and precuneus in the TLE group compared with the HC group (GRF corrected, *P*_voxel_ < 0.005, *P*_*cluster*_ < 0.05; Figure 1F and Table 2).

For dVMHC, TLE decreased in bilateral CAL, cuneus, and paracentral lobule (PCL) (GRF corrected, *P*_voxel_ < 0.005, *P*_*cluster*_ < 0.05; Figure 2F and Table 2).

#### Intergroup differences between left temporal lobe epilepsy and right temporal lobe epilepsy

We have performed a statistical analysis of the aforementioned static and dynamic IBA indicators between the LTLE (30 patients) and RTLE groups (27 patients). However, no significant cluster was found between LTLE and RTLE groups after multiple comparison correction (GRF corrected, *P*_voxel_ < 0.005, *P*_*cluster*_ < 0.05).

### Correlation analyses

As depicted in Table 3, significant correlations were detected between the values of ALFF, fALFF, ReHo, DC, GSCorr, VMHC, dALFF, dfALFF, dReHo, dDC, and dGSCorr in abnormal brain regions and several cognitive scales scores (*P* < 0.05). Moreover, most of them significantly correlated with the duration of epilepsy (*P* < 0.05). However, no significant correlation was observed between dVMHC and duration of epilepsy, NHS3, or cognitive scales scores (*P* > 0.05).

**TABLE 3:** Correlations between the values of IBA indicators in abnormal brain regions and epilepsy duration, NHS3, or cognitive scales scores.

Brain regions		Epilepsy duration	NHS3	MMSE	MES	MOCA	N1	N2	N3	N4	N5	Reconfiguration	STT-A	STT-B
**ALFF**														
MCG_L	r	0.319	−	−		−	−	−	−	−	−	−	−	−
	P	0.008	−	−		−	−	−	−	−	−	−	−	−
Cerebullum 6_L	r	0.315	−	−		–0.243	−	−	−	−	−	−	0.280	0.237
	P	0.008	−	−		0.034	−	−	−	−	−	−	0.017	0.038
Hippocampus_L	r	0.286	0.230	−		−	−	−	−	−	−	−	0.229	0.235
	P	0.016	0.043	−		−	−	−	−	−	−	−	0.043	0.039
SMA_L	r	−	−	−		−	−	−	−	−	−	−	–0.310	–0.245
	P	−	−	−		−	−	−	−	−	−	−	0.010	0.033
Precuneus_L	r	−	−	0.373		0.234	0.247	−	0.254	0.262	0.267	0.369	–0.324	−
	P	−	−	0.002		0.040	0.032	−	0.028	0.024	0.023	0.002	0.007	−
Pallidum_R	r	0.504	−	−		–0.260	−	−	−	−	−	−	0.229	0.235
	P	0.000	−	−		0.025	−	−	−	−	−	−	0.043	0.039
PostCG_R	r	–0.244	−	−		−	−	−	−	−	−	−	−	–0.273
	P	0.034	−	−		−	−	−	−	−	−	−	−	0.020
OFG_R	r	–0.280	−	−		−	−	−	–0.272	−	−	–0.246	−	−
	P	0.017	−	−		−	−	−	0.020	−	−	0.033	−	−
dALFF														
Cerebellum 4_5_L	r	0.251		−	−	−		−	−		−	−	−	−
	P	0.030		−	−	−		−	−		−	−	−	−
Thalamus_L	r	0.330		−	–0.246	−		−	−		−	−	0.419	0.374
	P	0.006		−	0.032	−		−	−		−	−	0.001	0.002
Thalamus_R	r	0.389		−	−	–0.223		−	−		−	−	0.275	0.268
	P	0.001		−	−	0.048		−	−		−	−	0.019	0.022
Cerebellum Crus1_R	r	–0.275		−	−	−		−	–0.266		−	−	−	−
	P	0.019		−	−	−		−	0.023		−	−	−	−
fALFF														
Hippocampus_R	r	0.356	−	–0.297		–0.255	−	−	−	−	−	–0.227	0.226	−
	P	0.003	−	0.013		0.028	−	−	−	−	−	0.045	0.045	−
Hippocampus_L	r	−	−	−		–0.280	−	−	−	−	−	−	0.261	0.256
	P	−	−	−		0.018	−	−	−	−	−	−	0.025	0.027
Thalamus_L	r	−	−	–0.306		−	−	−	−	−	−	−	0.232	−
	P	−	−	0.010		−	−	−	−	−	−	−	0.041	−
PostCG_R	r	–0.270	−	−		−	−	−	−	−	−	−	−	−
	P	0.021	−	−		−	−	−	−	−	−	−	−	−
dfALFF														
SMA_L	r	−		–0.297	−	−		−	−		−	0.222	−	−
	P	−		0.013	−	−		−	−		−	0.048	−	−
DSFG_R	r	0.238		−	0.229	0.259		−	0.317		0.267	0.309	–0.245	−
	P	0.037		−	0.043	0.026		−	0.008		0.022	0.010	0.033	−
ReHo														
MTG_L	r	–0.313	−	0.329		0.269	−	−	−	−	−	−	−	−
	P	0.009	−	0.006		0.022	−	−	−	−	−	−	−	−
SMA_L	r	−	−	−		−	−	−	−	−	−	−	–0.253	−
	P	−	−	−		−	−	−	−	−	−	−	0.029	−
dReHo														
CAL_R	r	–0.281		−	−	−		−	−		−	−	−	–0.267
	P	0.017		−	−	−		−	−		−	−	−	0.022
STG_L	r	−		−	−	−		−	–0.249		−	–0.256	0.255	−
	P	−		−	−	−		−	0.031		−	0.027	0.028	−
DC														
Precuneus_L	r	−	−	−		−	−	−	−	−	−	0.296	−	−
	P	−	−	−		−	−	−	−	−	−	0.013	−	−
SMA_L	r	−	−	−		−	−	−	−	−	−	−	–0.258	−
	P	−	−	−		−	−	−	−	−	−	−	0.026	−
dDC														
MTG_L	r	0.249		−	−	−		0.249	0.385		−	0.325	−	−
	P	0.031		−	−	−		0.031	0.002		−	0.007	−	−
CAL_L	r	–0.222		−	−	−		−	–0.223		−	–0.274	−	−
	P	0.048		−	−	−		−	0.047		−	0.019	−	−
CAL_R	r	−		−	−	−		−	–0.271		−	–0.270	−	−
	P	−		−	−	*v*		−	0.021		−	0.021	−	−
STG_L	r	−		−	−	−		−	–0.305		−	–0.297	−	−
	P	−		−	−	−		−	0.011		−	0.012	−	−
GSCorr														
ITG_L	r	−	−	−		−	0.264	0.341	0.306	0.292	0.359	0.307	−	−
	P	−	−	−		−	0.024	0.005	0.010	0.014	0.003	0.010	−	−
dGSCorr														
CAL_L	r	–0.232		−	−	−		−	–0.334		−	–0.408	−	−
	P	0.041		−	−	−		−	0.006		−	0.001	−	−
vSTG_L	r	−		−	−	−		−	–0.223		−	–0.308	−	−
	P	−		−	−	−		−	0.047		−	0.010	−	−
VMHC														
Bilateral MTG	r	–0.422	−	−		0.240	−	−	−	−	−	−	−	−
	P	0.001	−	−		0.036	−	−	−	−	−	−	−	−
Bilateral Cerebellum 6	r	–0.463	−	−		−	−	−	−	−	−	−	−	−
	P	0.000	−	−		−	−	−	−	−	−	−	−	−
Bilateral Precuneus	r	–0.222	−	−		−	−	−	−	−	−	−	−	–0.243
	P	0.049	−	−		−	−	−	−	−	−	−	−	0.034

ALFF, amplitude of low-frequency fluctuation; CAL_R, right calcarine cortex; dALFF, dynamic fractional ALFF; DC, degree centrality; dDC, dynamic degree centrality; dfALFF, dynamic fractional ALFF; dGSCorr, dynamic global signal correlation; dReHo, dynamic regional homogeneity; DSFG_R, right dorsolateral superior frontal gyrus; fALFF, fractional ALFF; GSCorr, global signal correlation; IBA, intrinsic brain activity; ITG_L, left inferior temporal gyrus; MCG_L, left median cingulate gyrus; MES, memory and executive screening; MMSE, Mini-Mental State Examination; MOCA, Montreal cognitive assessment-basic; MTG_L, left middle temporal gyrus; NHS3, national hospital seizure severity scale; OFG_R, right orbital frontal gyrus; PostCG_R, right postcentral gyrus; ReHo, regional homogeneity; SMA_L, left supplementary motor area; STG_L, left superior temporal gyrus, STT-A, shape trail making test-A; STT-B, shape trail making-B; VMHC.

### Validation analyses

When other window sizes/step sizes (60/10 TRs and 80/10 TRs) were used to validate the findings of the dynamic IBA indicators, the results were basically consistent with our main results.

## Discussion

We identified alteration of IBA in patients with TLE using static and dynamic ALFF, fALFF, ReHo, DC, VMHC, and GSCorr in TLE and HC groups. A large amount of overlap existed among the abnormal brain regions detected using various indicators. The convergence of the findings might suggest the existence of a more fundamental mechanism capable of revealing more real abnormal neuronal activity in TLE other than specific effects caused by particular imaging algorithms.

### Regions displaying increased amplitude of low-frequency fluctuation/dynamic amplitude of low-frequency fluctuation/fractional amplitude of low-frequency fluctuation and “temporal epilepsy network”

In our study, brain regions displaying considerable differences overlapped in ALFF, dALFF, and fALFF, which increased in the bilateral medial temporal lobe (MTL) and thalamus. Epileptic seizures could initiate a neural circuit and lead to aberrant neural communication with brain areas outside the epileptogenic region. MTL and thalamus were reported to compose a temporal epilepsy network proposed previously, which were involved in the initiation, propagation, and facilitation of epileptic activity (Spencer, 2002). An increase in ALFF in these regions has been reported in the study by Zhang et al. (2010). Moreover, the widespread increases in ALFF/dALFF or fALFF in various cortical and subcortical structures (cerebellum, CG, precuneus, SMA, and pons ipsilateral to the epileptogenic foci) besides the MTL and thalamus might also implicate the pathologic substrates of an epileptic network in TLE (Schacher et al., 2006).

The ALFF changes in the BOLD signal have been suggested to be associated with local neuronal activity (Logothetis et al., 2001; Yang et al., 2007) and interictal epileptic discharges (IEDs) (Zhang et al., 2010). fALFF represents the ratio of the low-frequency power spectrum to that of the entire frequency range, which could effectively suppress the physiologic noise (Zou et al., 2008). Liao et al. (2019) demonstrated that the electrophysiologic relevance of ALFF variability depended on EEG power fluctuations, suggesting a neurophysiologic basis. Thus, dALFF was supposed to represent the temporal variability of IBA fluctuation, and higher dALFF represented greater volatility and instability of the neuronal activity. The increase in ALFF, fALFF, and dALFF indicated that epileptic activity might not only induce hyperactivity of neuronal activity in the epilepsy network but also cause an abnormal unstable state of neuronal activity, further leading to a chaotic state of brain function.

Medial temporal lobe structures, such as the hippocampus, PHG, and amygdala, are involved in memory, learning, and emotion regulation (Burwell, 2000; Viard et al., 2007). Excessive but unstable brain activity in the hippocampus and PHG may lead to the failure to accomplish learning and memory processes efficiently. The hyperactivity of the amygdala may contribute to the fear aura, emotional lability, and temperament change. Thalamus was reported to be a relay center in a complex reciprocal network linking cortices and other subcortical structures (Behrens et al., 2003; Blumenfeld et al., 2004) and responsible for information integration and executive functioning (Tuchscherer et al., 2010). Our correlation analysis results confirmed that increased ALFF, fALFF, or dALFF in MTL and thalamus was related to longer epilepsy duration and more serious cognition decline. These results suggested that TLE, as a persisting disease, revealed a gradually aggravated effect on the brain activity and cognition of patients. Considering the thalamus as an example, the increased fALFF and dALFF in the thalamus negatively correlated with MMSE and MES/MOCA, respectively. In addition, increased fALFF and dALFF both negatively correlated with the time consumption of STT-A/B. These indicated that excessive but unstable activity in the thalamus was related to instability of the information transmission and further execution or cognition impairment.

### Regions displaying decreased amplitude of low-frequency fluctuation/dynamic amplitude of low-frequency fluctuation/fractional amplitude of low-frequency fluctuation and “network inhibition hypothesis”

In our study, regions displaying decreased ALFF, fALFF, or dALFF, mainly located in the frontal lobe contralateral to the epileptogenic side, overlapped with the regions of PreCG, PostCG, IFG, MFG, and OFG. The sensorimotor network (SMN) was mainly composed of PreCG, PostCG, and SMA. The decrease in ALFF and fALFF in SMN might represent the decline or inhibition of brain activity and lead to a series of motor symptoms or disorders caused by epilepsy (Laufs et al., 2014). Moreover, as an advanced cognitive center, the frontal lobe was also essential for the language, execution, and control functions (Rosch and Mostofsky, 2019) and was a component of the cognitive-attention network (Bush, 2011). OFG could adaptively shape decision-making and affective behavior (Rudebeck and Rich, 2018). As a structure related to epileptic activity propagation, the frontal lobe could be highly affected. Its dysfunction in TLE has been affirmed by behavioral tests (Hermann et al., 1991), fALFF based on rs-fMRI (Reyes et al., 2016), as well as PET (Guedj et al., 2015) and SPECT (Blumenfeld et al., 2004) imaging.

Similar to these studies, our results indicated that the decreasing ALFF and fALFF values in the contralateral PostCG negatively correlated with epilepsy duration and STT-B, suggesting that the activity decline in PostCG was associated with the impairment of execution-control functions and cognitive flexibility, which was reflected by STT, and worsened with prolonged epilepsy duration. According to the “network inhibition hypothesis” of TLE proposed by Blumenfeld et al. (2004), (Norden and Blumenfeld, 2002; Blumenfeld and Taylor, 2003), we cautiously speculated that the negative activation changes in ALFF, fALFF, and dALFF in the frontal lobe further confirmed the existence of the inhibition network. On the one hand, the negative activation represented functional inactivation, which would lead to cognition decline and loss of consciousness. On the other hand, it might represent an inhibitory effect of the brain on epileptic activity.

### Decreased fractional amplitude of low-frequency fluctuation, regional homogeneity, dynamic regional homogeneity, degree centrality, dynamic degree centrality, global signal correlation, dynamic global signal correlation, and voxel-mirrored homotopic connectivity in the temporal neocortex ipsilateral to the epileptogenic foci

Our study demonstrated that various static and dynamic IBA indicators, including fALFF, ReHo, dReHo, DC, dDC, GSCorr, dGSCorr, and VMHC, decreased in the temporal neocortex ipsilateral to the epileptogenic foci. A previous VMHC study verified the decisive role of the temporal lobe in distinguishing TLE from HC (Gao et al., 2021). These highly consistent results suggested a clinical potential for the disease classification and lateralization of TLE using IBA indicators.

The temporal neocortex included the structure of STG, MTG, ITG, TP, and Heschl’s gyrus, and was related to auditory function, language, memory, cognitive function, and emotion (Olson et al., 2007). Primary and association auditory cortices (Brodmann 41 and 42) and partial Wernicke’s area were located at STG; they were responsible for auditory and language information processing and also memory and cognitive function (Fletcher et al., 1995). The dysfunction of the auditory cortex might induce acousma and impairment of auditory-related cognition. MTG (Kiehl et al., 2004) and ITG (Dien et al., 2013) were also thought to be the key regions for language processing and formulation. Accessory visual areas (Brodmann 20 and 21), which were related to visual perception, were located at MTG and ITG, respectively. Moreover, the ITG was a key node in the broad network of frontal, temporal, parietal, occipital, and subcortical structures. Numerous studies demonstrated that fMRI revealed abnormality of the temporal neocortex and its correlation with cognitive function (Vaughan et al., 2016; Trimmel et al., 2018; Gao et al., 2021).

Similarly, our correlation analysis results further demonstrated that the abnormal IBA of the temporal neocortex was associated with multiple cognitive scores and duration of epilepsy. The decreasing ReHo and VMHC in MTG negatively correlated with epilepsy duration but positively correlated with MMSE or MOCA. The GSCorr in ITG positively correlated with AVLT. These findings indicated that the weakness of local and global function in the temporal neocortex was a crucial feature in patients with unilateral TLE, and might be responsible for the impairment of memory and cognitive function, which would deteriorate with time.

However, during the correlation assessment of dynamic indicators, we demonstrated that the decreasing dReHo, dDC, and dGSCorr were negatively correlated with AVLT. This might seem contrary to the aforementioned speculation, but in fact, the lower temporal variability represented a relatively more stable functional status, which we considered to reflect the existence of a “decompensatory mechanism” (Yang et al., 2018; Li et al., 2020; Singh et al., 2020). The existence of functional compensation has been reported in previous fMRI research (Yang et al., 2018; Li et al., 2020; Singh et al., 2020). It might improve the patients’ memory to a certain extent but could not replace the original functions effectively and might cause other functional disorders such as the decline in cognitive flexibility due to the low temporal variability in our study.

According to the implication represented by each indicator, we speculated that the dysfunction in the temporal neocortex was reflected not only in a decline of IBA intensity but also in reduced functional connectivity among neighboring regions, interhemispheric homotopic regions, and the remaining brain regions. Thus, large clusters of neurons were involved in inducing great interference in global brain function networks. The highly consistent decreased activation of static and dynamic IBA indicators might indicate a more fundamental regulatory mechanism in TLE, which we prudently speculated to be the “surround inhibition hypothesis” (Schwartz and Bonhoeffer, 2001; Van Paesschen et al., 2003; Blumenfeld et al., 2004). It was hypothesized that the regions surrounding the epileptogenic focus might serve an adaptive function to cut down the possibility of seizure spread to other regions. Actually, TLE was mostly confined to the limbic cortex and rarely generalized. Combined with the aforementioned decreased activation of the frontal lobe, we further speculated that the temporal neocortex was also the component of the TLE inhibition network.

### Decreased dynamic regional homogeneity, dynamic degree centrality, dynamic global signal correlation, and dynamic voxel-mirrored homotopic connectivity in the occipital lobe

In our study, the most impressive result of dynamic IBA indicators was that dReho, dDC, dGSCorr, and dVMHC decreased in the bilateral occipital lobe. However, no abnormal changes in the occipital lobe were detected in the static indicators, indicating the complementarity of dynamic and static indicators. The temporal dynamic indicators were expected to become a novel and more powerful and sensitive imaging-related neurobiologic indicator.

Visual network (VIN) was part of the sensor cortex systems and mainly located in the occipital lobe, including CAL, LG, cuneus, and so forth. As the center of the visual cortex, the occipital lobe exhibited a range of visual functions, such as vision processing and visual memory encoding (Mechelli et al., 2000), and was related to executive function and attention (Ishai et al., 2002) because the acquisition of visual information was a prerequisite for attention. Memory and alertness function depend on sensory-perceptual information processing, such as vision and audition (Conway, 2001). Thus, the occipital lobe’s dysfunction was not merely related to a primary visual abnormality, such as visual hallucinations or auras, but also to high-order visual function and cognition impairment (Alessio et al., 2013).

Previous studies detected VIN abnormalities in patients with TLE (Zhang et al., 2009; Cataldi et al., 2013). Lu et al. (2018) reported a consistent decline in dynamic ReHo, DC, and ALFF in the occipital lobe of patients with autism. According to the implication represented by each static indicator, we speculated that the decreases in dReho, dVMHC, dDC, and dGSCorr suggested a lack of essential flexibility and plasticity in functional connectivity among neighboring regions, interhemispheric homotopic regions, and the remaining brain regions. We speculated that inflexible IBA in the visual processing brain areas might lead to difficulty in instant and efficient management of visual information, short-term memory, and alertness. The STT-B mainly evaluated the flexibility of cognitive function based on the sensitivity and working speed of visual perception. Our results indicated that the decreasing dReHo values in CAL negatively correlated with STT-B, which confirmed our speculation. Besides, the epilepsy duration negatively correlated with dReHo, dDC, and dGSCorr, indicating that the IBA flexibility worsened with prolonged epilepsy duration.

Nevertheless, we demonstrated that the decreasing dDC and dGSCorr negatively correlated with AVLT, which seemed to contradict our speculation. However, as AVLT emphasized auditory and verbal memory, we speculated that this phenomenon might be related to the “decompensation mechanism” (Yang et al., 2018; Li et al., 2020; Singh et al., 2020) occurring in VIN after the severe dysfunction of the temporal neocortex in patients with TLE.

### Increased amplitude of low-frequency fluctuation, fractional amplitude of low-frequency fluctuation, dynamic fractional amplitude of low-frequency fluctuation, regional homogeneity, and degree centrality in supplementary motor area ipsilateral to the epileptogenic foci

Our study demonstrated that ALFF, fALFF, dfALFF, ReHo, and DC all increased in SMA ipsilateral to the epileptogenic foci. SMA was a vital motor cortex belonging to the sensorimotor network and could evoke spasticity and cortical motor aphasia (Rostomily et al., 1991; Trimmel et al., 2018). SMA plays an indispensable role in the “intention-action” process (Kalaska, 2009), and participates in the preparation, initiation, and monitoring of complex movements (Picard and Strick, 1996; Michelon et al., 2006). Several fMRI studies have reported the functional changes in the SMA elicited by epilepsy (O’Muircheartaigh et al., 2012; Laufs et al., 2014; Morgan et al., 2020). It was suggested that SMA was responsible for motor dysfunction in patients with epilepsy and positively responded to drug treatment (Morgan et al., 2020).

The increases in ALFF, fALFF, and dfALFF represent a high amplitude and excessive unstable state of IBA due to epileptic activity. Based on this, we speculated that SMA ipsilateral to the epileptogenic foci was a crucial constituent of the TLE network and was related to the propagation and facilitation of epileptic activity. The increased ReHo and DC in SMA further verified this speculation. Similarly, the increased activation of ALFF and ReHo in SMA has been reported earlier (Zhang et al., 2010).

The correlation results revealed that the dfALFF and MMSE negatively correlated, indicating that the over-high temporal variability in SMA might affect the precise and stable execution of higher-order motor and cognitive processes, further inducing cognition decline.

### Limitations

The present study had several limitations. Firstly, the sample size was small, and all participants came from a single dataset. Studies involving larger samples or another independent dataset are required in the future to confirm our findings. Secondly, the present study compared only the differences between patients with right/left flipped LTLE and HCs, but the differences among patients with LTLE and RTLE and HCs were not analyzed directly due to the small sample size. Previous studies have shown several brain activity measurements’ differences between LTLE and RTLE. Zhang et al. (2010) and Amiri et al. (2020) have separately demonstrated that the utility of ALFF analysis and graph theory application to brain network analysis might be a potential biomarker to assist in the determination of TLE laterality. Pang et al. (Li et al., 2022) found that RTLE patients exhibited more pronounced aberrant connectivity patterns and topological properties revealed by static or dynamic functional connectivity. Consequently, a subgroup analysis is necessary in the future study. Thirdly, further studies on the other IBA indicators, such as FC and ICA, etc., are needed to validate the results, especially the detailed functional connectivity between abnormally activated brain regions and other regions. Fourthly, we did not monitor the numbers or frequency of the epileptic spike during the fMRI scan period simultaneously. And cause some of the IBA indicators might be affected by the epileptic spikes, so the application of simultaneous EEG/fMRI in the future study was important. Fifthly, about the sliding window technique that we have performed, the question about the choice of window width has been constantly discussed, tried and verified. So the application of other dynamic assessment methods (Chen et al., 2017) to verify our results are necessary in the future.

## Conclusion

We comprehensively estimated the differences in IBA between patients with TLE and HC using multiple novel static/dynamic indicators, as well as their correlation with cognition. IBA abnormalities in patients with TLE were detected in multiple brain network–related regions, including the temporal lobe epilepsy network (MTL structure and thalamus), sensorimotor network (SMA, PreCG, and PostCG), auditory network (temporal neocortex), and visual network (occipital lobe). The combined application of static and dynamic IBA indicators could comprehensively reveal more real abnormal neuronal activity and the impairment and compensatory mechanisms of cognitive function in TLE. Moreover, it might help in the lateralization of epileptogenic foci and exploration of the transmission and inhibition pathways of epileptic activity.

## Ethics statement

The studies involving human participants were reviewed and approved by the Research Ethics Committee at The First Affiliated Hospital of Zhengzhou University. Written informed consent to participate in this study was provided by the participants’ legal guardian/next of kin.

## Author contributions

CS wrote the first draft of the manuscript. CS and XNZ performed the data and statistical analysis. SH provided the methodological advice. YL provided the clinical diagnosis of the participants. CS, XNZ, KM, KW, and XM collected the data. XCZ and JZ provided the technical advice and proofread the manuscript. JC and YZ contributed to the design of the study and proofread the manuscript. All authors have read and agreed to the published version of the manuscript.

## References

[B1] Alessio A. Pereira F. R. Sercheli M. S. Rondina J. M. Ozelo H. B. Bilevicius E. (2013). Brain plasticity for verbal and visual memories in patients with mesial temporal lobe epilepsy and hippocampal sclerosis: An fMRI study. *Hum. Brain Mapp.* 34 186–199. 10.1002/hbm.21432 22038783 PMC6870348

[B2] Allone C. Lo Buono V. Corallo F. Pisani L. R. Pollicino P. Bramanti P. (2017). Neuroimaging and cognitive functions in temporal lobe epilepsy: A review of the literature. *J. Neurol. Sci.* 381 7–15. 10.1016/j.jns.2017.08.007 28991719

[B3] Amiri S. Mehvari-Habibabadi J. Mohammadi-Mobarakeh N. Hashemi-Fesharaki S. S. Mirbagheri M. M. Elisevich K. (2020). Graph theory application with functional connectivity to distinguish left from right temporal lobe epilepsy. *Epilepsy Res.* 167:106449. 10.1016/j.eplepsyres.2020.106449 32937221

[B4] Araújo D. Santos A. C. Velasco T. R. Wichert-Ana L. Terra-Bustamante V. C. Alexandre V. Jr. (2006). Volumetric evidence of bilateral damage in unilateral mesial temporal lobe epilepsy. *Epilepsia.* 47 1354–1359. 10.1111/j.1528-1167.2006.00605.x 16922881

[B5] Beckmann C. F. DeLuca M. Devlin J. T. Smith S. M. (2005). Investigations into resting-state connectivity using independent component analysis. *Philos. Trans. R. Soc. Lond. B Biol. Sci.* 360 1001–1013. 10.1098/rstb.2005.1634 16087444 PMC1854918

[B6] Behrens T. E. Johansen-Berg H. Woolrich M. W. Smith S. M. Wheeler-Kingshott C. A. Boulby P. A. (2003). Non-invasive mapping of connections between human thalamus and cortex using diffusion imaging. *Nat. Neurosci.* 6 750–757. 10.1038/nn1075 12808459

[B7] Bernasconi N. Bernasconi A. Caramanos Z. Antel S. B. Andermann F. Arnold D. L. (2003). Mesial temporal damage in temporal lobe epilepsy: A volumetric MRI study of the hippocampus, amygdala and parahippocampal region. *Brain* 126(Pt 2), 462–469. 10.1093/brain/awg034 12538412

[B8] Bettus G. Wendling F. Guye M. Valton L. Régis J. Chauvel P. (2008). Enhanced EEG functional connectivity in mesial temporal lobe epilepsy. *Epilepsy Res.* 81 58–68. 10.1016/j.eplepsyres.2008.04.020 18547787

[B9] Biswal B. Yetkin F. Z. Haughton V. M. Hyde J. S. (1995). Functional connectivity in the motor cortex of resting human brain using echo-planar MRI. *Magn. Reson. Med.* 34 537–541. 10.1002/mrm.1910340409 8524021

[B10] Blumenfeld H. McNally K. A. Vanderhill S. D. Paige A. L. Chung R. Davis K. (2004). Positive and negative network correlations in temporal lobe epilepsy. *Cereb. Cortex* 14 892–902. 10.1093/cercor/bhh048 15084494

[B11] Blumenfeld H. Taylor J. (2003). Why do seizures cause loss of consciousness? *Neuroscientist* 9 301–310. 10.1177/1073858403255624 14580115

[B12] Blumenfeld H. Varghese G. I. Purcaro M. J. Motelow J. E. Enev M. McNally K. A. (2009). Cortical and subcortical networks in human secondarily generalized tonic-clonic seizures. *Brain* 132(Pt 4), 999–1012. 10.1093/brain/awp028 19339252 PMC2724910

[B13] Burianová H. Faizo N. L. Gray M. Hocking J. Galloway G. Reutens D. (2017). Altered functional connectivity in mesial temporal lobe epilepsy. *Epilepsy Res.* 137 45–52. 10.1016/j.eplepsyres.2017.09.001 28923408

[B14] Burwell R. D. (2000). The parahippocampal region: Corticocortical connectivity. *Ann. N. Y. Acad. Sci.* 911 25–42. 10.1111/j.1749-6632.2000.tb06717.x 10911865

[B15] Bush G. (2011). Cingulate, frontal, and parietal cortical dysfunction in attention-deficit/hyperactivity disorder. *Biol. Psychiatry* 69 1160–1167. 10.1016/j.biopsych.2011.01.022 21489409 PMC3109164

[B16] Cataldi M. Avoli M. de Villers-Sidani E. (2013). Resting state networks in temporal lobe epilepsy. *Epilepsia* 54 2048–2059. 10.1111/epi.12400 24117098 PMC4880458

[B17] Chao-Gan Y. Yu-Feng Z. (2010). DPARSF: A MATLAB toolbox for “Pipeline” data analysis of resting-state fMRI. *Front. Syst. Neurosci.* 4:13. 10.3389/fnsys.2010.00013 20577591 PMC2889691

[B18] Chen J. E. Rubinov M. Chang C. (2017). Methods and considerations for dynamic analysis of functional MR imaging data. *Neuroimaging Clin. N. Am.* 27 547–560. 10.1016/j.nic.2017.06.009 28985928 PMC5679015

[B19] Concha L. Beaulieu C. Gross D. W. (2005). Bilateral limbic diffusion abnormalities in unilateral temporal lobe epilepsy. *Ann. Neurol.* 57 188–196. 10.1002/ana.20334 15562425

[B20] Conway M. A. (2001). Sensory-perceptual episodic memory and its context: Autobiographical memory. *Philos. Trans. R. Soc. Lond. B Biol. Sci.* 356 1375–1384. 10.1098/rstb.2001.0940 11571029 PMC1088521

[B21] Dien J. Brian E. S. Molfese D. L. Gold B. T. (2013). Combined ERP/fMRI evidence for early word recognition effects in the posterior inferior temporal gyrus. *Cortex* 49 2307–2321. 10.1016/j.cortex.2013.03.008 23701693 PMC3758432

[B22] Engel J. Jr. (2001). International League Against Epilepsy (ILAE). A proposed diagnostic scheme for people with epileptic seizures and with epilepsy: Report of the ILAE Task Force on Classification and Terminology. *Epilepsia* 42 796–803. 10.1046/j.1528-1157.2001.10401.x 11422340

[B23] Fletcher P. C. Frith C. D. Grasby P. M. Shallice T. Frackowiak R. S. Dolan R. J. (1995). Brain systems for encoding and retrieval of auditory-verbal memory. An in vivo study in humans. *Brain* 118(Pt 2), 401–416. 10.1093/brain/118.2.401 7735882

[B24] Fox M. D. Raichle M. E. (2007). Spontaneous fluctuations in brain activity observed with functional magnetic resonance imaging. *Nat. Rev. Neurosci.* 8 700–711. 10.1038/nrn2201 17704812

[B25] Gao Y. J. Wang X. Xiong P. G. Ren H. W. Zhou S. Y. Yan Y. G. (2021). Abnormalities of the default-mode network homogeneity and executive dysfunction in people with first-episode, treatment-naive left temporal lobe epilepsy. *Eur. Rev. Med. Pharmacol. Sci.* 25 2039–2049. 10.26355/eurrev_202102_25108 33660816

[B26] Gonzalez-Castillo J. Hoy C. W. Handwerker D. A. Robinson M. E. Buchanan L. C. Saad Z. S. (2015). Tracking ongoing cognition in individuals using brief, whole-brain functional connectivity patterns. *Proc. Natl. Acad. Sci. U.S.A.* 112 8762–8767. 10.1073/pnas.1501242112 26124112 PMC4507216

[B27] Guedj E. Bonini F. Gavaret M. Trébuchon A. Aubert S. Boucekine M. (2015). 18FDG-PET in different subtypes of temporal lobe epilepsy: SEEG validation and predictive value. *Epilepsia* 56 414–421. 10.1111/epi.12917 25708545

[B28] Hermann B. P. Seidenberg M. Haltiner A. Wyler A. R. (1991). Mood state in unilateral temporal lobe epilepsy. *Biol. Psychiatry* 30 1205–1218. 10.1016/0006-3223(91)90157-h 1790262

[B29] Hindriks R. Adhikari M. H. Murayama Y. Ganzetti M. Mantini D. Logothetis N. K. (2016). Corrigendum to “Can sliding-window correlations reveal dynamic functional connectivity in resting-state fMRI?” [NeuroImage 127 (2016) 242-256]. *Neuroimage* 132:115. 10.1016/j.neuroimage.2016.02.007 26631813 PMC4758830

[B30] Holmes M. Folley B. S. Sonmezturk H. H. Gore J. C. Kang H. Abou-Khalil B. (2014). Resting state functional connectivity of the hippocampus associated with neurocognitive function in left temporal lobe epilepsy. *Hum. Brain Mapp.* 35 735–744. 10.1002/hbm.22210 23124719 PMC3915042

[B31] Ishai A. Haxby J. V. Ungerleider L. G. (2002). Visual imagery of famous faces: Effects of memory and attention revealed by fMRI. *Neuroimage* 17 1729–1741. 10.1006/nimg.2002.1330 12498747

[B32] Jiang L. W. Qian R. B. Fu X. M. Zhang D. Peng N. Niu C. S. (2018). Altered attention networks and DMN in refractory epilepsy: A resting-state functional and causal connectivity study. *Epilepsy Behav.* 88 81–86. 10.1016/j.yebeh.2018.06.045 30243110

[B33] Jiang S. Luo C. Huang Y. Li Z. Chen Y. Li X. (2020). Altered static and dynamic spontaneous neural activity in drug-naïve and drug-receiving benign childhood epilepsy with centrotemporal spikes. *Front. Hum. Neurosci.* 14:361. 10.3389/fnhum.2020.00361 33005141 PMC7485420

[B34] Kalaska J. F. (2009). From intention to action: Motor cortex and the control of reaching movements. *Adv. Exp. Med. Biol.* 629 139–178. 10.1007/978-0-387-77064-2_8 19227499

[B35] Keezer M. R. Sisodiya S. M. Sander J. W. (2016). Comorbidities of epilepsy: Current concepts and future perspectives [published correction appears in Lancet Neurol. 2016 Jan;15(1):28]. *Lancet Neurol.* 15 106–115. 10.1016/S1474-4422(15)00225-2 26549780

[B36] Kiehl K. A. Smith A. M. Mendrek A. Forster B. B. Hare R. D. Liddle P. F. (2004). Temporal lobe abnormalities in semantic processing by criminal psychopaths as revealed by functional magnetic resonance imaging. *Psychiatry Res.* 130 297–312. 10.1016/j.pscychresns.2004.02.002 15209063

[B37] Laufs H. Rodionov R. Thornton R. Duncan J. S. Lemieux L. Tagliazucchi E. (2014). Altered FMRI connectivity dynamics in temporal lobe epilepsy might explain seizure semiology. *Front. Neurol.* 5:175. 10.3389/fneur.2014.00175 25309503 PMC4160997

[B38] Li H. Ding F. Chen C. Huang P. Xu J. Chen Z. (2022). Dynamic functional connectivity in modular organization of the hippocampal network marks memory phenotypes in temporal lobe epilepsy. *Hum. Brain Mapp.* 43 1917–1929. 10.1002/hbm.25763 34967488 PMC8933317

[B39] Li R. Hu C. Wang L. Liu D. Liu D. Liao W. (2020). Disruption of functional connectivity among subcortical arousal system and cortical networks in temporal lobe epilepsy. *Brain Imaging Behav.* 14 762–771. 10.1007/s11682-018-0014-y 30617780

[B40] Liang X. Pang X. Liu J. Zhao J. Yu L. Zheng J. (2020). Comparison of topological properties of functional brain networks with graph theory in temporal lobe epilepsy with different duration of disease. *Ann. Transl. Med.* 8:1503. 10.21037/atm-20-6823 33313248 PMC7729351

[B41] Liao W. Li J. Ji G. J. Wu G. R. Long Z. Xu Q. (2019). Endless fluctuations: Temporal dynamics of the amplitude of low frequency fluctuations. *IEEE Trans. Med. Imaging* 38 2523–2532. 10.1109/TMI.2019.2904555 30872224

[B42] Liao W. Wu G. R. Xu Q. Ji G. J. Zhang Z. Zang Y. F. (2014). DynamicBC: A MATLAB toolbox for dynamic brain connectome analysis. *Brain Connect.* 4 780–790. 10.1089/brain.2014.0253 25083734 PMC4268585

[B43] Logothetis N. K. Pauls J. Augath M. Trinath T. Oeltermann A. (2001). Neurophysiological investigation of the basis of the fMRI signal. *Nature* 412 150–157. 10.1038/35084005 11449264

[B44] Lu B. Chen X. Li L. Shen Y. Q. Chen N. X. Mei T. (2018). Aberrant dynamics of spontaneous brain activity and its integration in patients with autism spectrum disorder. *Chin. Sci. Bull.* 63 1452–1463.

[B45] Maneshi M. Vahdat S. Fahoum F. Grova C. Gotman J. (2014). Specific resting-state brain networks in mesial temporal lobe epilepsy. *Front. Neurol.* 5:127. 10.3389/fneur.2014.00127 25071712 PMC4095676

[B46] Margulies D. S. Böttger J. Long X. Lv Y. Kelly C. Schäfer A. (2010). Resting developments: A review of fMRI post-processing methodologies for spontaneous brain activity. *MAGMA* 23 289–307. 10.1007/s10334-010-0228-5 20972883

[B47] Mechelli A. Humphreys G. W. Mayall K. Olson A. Price C. J. (2000). Differential effects of word length and visual contrast in the fusiform and lingual gyri during reading. *Proc. Biol. Sci.* 267 1909–1913. 10.1098/rspb.2000.1229 11052544 PMC1690747

[B48] Michelon P. Vettel J. M. Zacks J. M. (2006). Lateral somatotopic organization during imagined and prepared movements. *J. Neurophysiol.* 95 811–822. 10.1152/jn.00488.2005 16207787

[B49] Morgan V. L. Chang C. Englot D. J. Rogers B. P. (2020). Temporal lobe epilepsy alters spatio-temporal dynamics of the hippocampal functional network. *Neuroimage Clin.* 26:102254. 10.1016/j.nicl.2020.102254 32251905 PMC7132094

[B50] Nakai Y. Nishibayashi H. Donishi T. Terada M. Nakao N. Kaneoke Y. (2021). Regional abnormality of functional connectivity is associated with clinical manifestations in individuals with intractable focal epilepsy. *Sci. Rep.* 11:1545. 10.1038/s41598-021-81207-6 33452388 PMC7810833

[B51] Norden A. D. Blumenfeld H. (2002). The role of subcortical structures in human epilepsy. *Epilepsy Behav.* 3 219–231. 10.1016/s1525-5050(02)00029-x 12662601

[B52] Olson I. R. Plotzker A. Ezzyat Y. (2007). The Enigmatic temporal pole: A review of findings on social and emotional processing. *Brain* 130(Pt 7), 1718–1731. 10.1093/brain/awm052 17392317

[B53] O’Muircheartaigh J. Vollmar C. Barker G. J. Kumari V. Symms M. R. Thompson P. (2012). Abnormal thalamocortical structural and functional connectivity in juvenile myoclonic epilepsy. *Brain* 135(Pt 12), 3635–3644. 10.1093/brain/aws296 23250883 PMC3525058

[B54] Pang X. Liang X. Zhao J. Wu P. Li X. Wei W. (2022). Abnormal static and dynamic functional connectivity in left and right temporal lobe epilepsy. *Front. Neurosci.* 15:820641. 10.3389/fnins.2021.820641 35126048 PMC8813030

[B55] Park H. J. Friston K. J. Pae C. Park B. Razi A. (2018). Dynamic effective connectivity in resting state fMRI. *Neuroimage* 180(Pt B), 594–608. 10.1016/j.neuroimage.2017.11.033 29158202 PMC6138953

[B56] Picard N. Strick P. L. (1996). Motor areas of the medial wall: A review of their location and functional activation. *Cereb. Cortex* 6 342–353. 10.1093/cercor/6.3.342 8670662

[B57] Power J. D. Barnes K. A. Snyder A. Z. Schlaggar B. L. Petersen S. E. (2012). Spurious but systematic correlations in functional connectivity MRI networks arise from subject motion. *Neuroimage* 59 2142–2154. 10.1016/j.neuroimage.2011.10.018 22019881 PMC3254728

[B58] Power J. D. Plitt M. Laumann T. O. Martin A. (2017). Sources and implications of whole-brain fMRI signals in humans. *Neuroimage* 146 609–625. 10.1016/j.neuroimage.2016.09.038 27751941 PMC5321814

[B59] Raichle M. E. (2010). Two views of brain function. *Trends Cogn. Sci.* 14 180–190. 10.1016/j.tics.2010.01.008 20206576

[B60] Raichle M. E. (2015). The restless brain: How intrinsic activity organizes brain function. *Philos. Trans. R. Soc. Lond. B Biol. Sci.* 370:20140172. 10.1098/rstb.2014.0172 25823869 PMC4387513

[B61] Reyes A. Thesen T. Wang X. Hahn D. Yoo D. Kuzniecky R. (2016). Resting-state functional MRI distinguishes temporal lobe epilepsy subtypes. *Epilepsia* 57 1475–1484. 10.1111/epi.13456 27374869

[B62] Rosch K. S. Mostofsky S. (2019). Development of the frontal lobe. *Handb. Clin. Neurol.* 163 351–367. 10.1016/B978-0-12-804281-6.00019-7 31590741

[B63] Rostomily R. C. Berger M. S. Ojemann G. A. Lettich E. (1991). Postoperative deficits and functional recovery following removal of tumors involving the dominant hemisphere supplementary motor area. *J. Neurosurg.* 75 62–68. 10.3171/jns.1991.75.1.0062 2045920

[B64] Rudebeck P. H. Rich E. L. (2018). Orbitofrontal cortex. *Curr. Biol.* 28 R1083–R1088. 10.1016/j.cub.2018.07.018 30253144 PMC9253859

[B65] Sakoğlu U. Pearlson G. D. Kiehl K. A. Wang Y. M. Michael A. M. Calhoun V. D. (2010). A method for evaluating dynamic functional network connectivity and task-modulation: Application to schizophrenia. *MAGMA* 23 351–366. 10.1007/s10334-010-0197-8 20162320 PMC2891285

[B66] Schacher M. Winkler R. Grunwald T. Kraemer G. Kurthen M. Reed V. (2006). Mesial temporal lobe epilepsy impairs advanced social cognition. *Epilepsia* 47 2141–2146. 10.1111/j.1528-1167.2006.00857.x 17201715

[B67] Schwartz T. H. Bonhoeffer T. (2001). In vivo optical mapping of epileptic foci and surround inhibition in ferret cerebral cortex. *Nat. Med.* 7 1063–1067. 10.1038/nm0901-1063 11533712

[B68] Shi K. Pang X. Wang Y. Li C. Long Q. Zheng J. (2021). Altered interhemispheric functional homotopy and connectivity in temporal lobe epilepsy based on fMRI and multivariate pattern analysis. *Neuroradiology* 63 1873–1882. 10.1007/s00234-021-02706-x 33938990

[B69] Singh T. B. Aisikaer A. He C. Wu Y. Chen H. Ni H. (2020). The assessment of brain functional changes in the temporal lobe epilepsy patient with cognitive impairment by resting-state functional magnetic resonance imaging. *J. Clin. Imaging Sci.* 10:50. 10.25259/JCIS_55_2020 PMC7451150 32874755

[B70] Spencer S. S. (2002). Neural networks in human epilepsy: Evidence of and implications for treatment. *Epilepsia* 43 219–227. 10.1046/j.1528-1157.2002.26901.x 11906505

[B71] Thijs R. D. Surges R. O’Brien T. J. Sander J. W. (2019). Epilepsy in adults. *Lancet* 393 689–701. 10.1016/S0140-6736(18)32596-0 30686584

[B72] Thompson G. J. Magnuson M. E. Merritt M. D. Schwarb H. Pan W. J. McKinley A. (2013). Short-time windows of correlation between large-scale functional brain networks predict vigilance intraindividually and interindividually. *Hum. Brain Mapp.* 34 3280–3298. 10.1002/hbm.22140 22736565 PMC6870033

[B73] Trimmel K. van Graan A. L. Caciagli L. Haag A. Koepp M. J. Thompson P. J. (2018). Left temporal lobe language network connectivity in temporal lobe epilepsy. *Brain* 141 2406–2418. 10.1093/brain/awy164 29939211

[B74] Tuchscherer V. Seidenberg M. Pulsipher D. Lancaster M. Guidotti L. Hermann B. (2010). Extrahippocampal integrity in temporal lobe epilepsy and cognition: Thalamus and executive functioning. *Epilepsy Behav.* 17 478–482. 10.1016/j.yebeh.2010.01.019 20185373

[B75] Van Paesschen W. Dupont P. Van Driel G. Van Billoen H. Maes A. (2003). SPECT perfusion changes during complex partial seizures in patients with hippocampal sclerosis. *Brain* 126(Pt 5), 1103–1111. 10.1093/brain/awg108 12690050

[B76] Vaughan D. N. Rayner G. Tailby C. Jackson G. D. (2016). MRI-negative temporal lobe epilepsy: A network disorder of neocortical connectivity. *Neurology* 87 1934–1942. 10.1212/WNL.0000000000003289 27694267

[B77] Viard A. Piolino P. Desgranges B. Chételat G. Lebreton K. Landeau B. (2007). Hippocampal activation for autobiographical memories over the entire lifetime in healthy aged subjects: An fMRI study. *Cereb. Cortex* 17 2453–2467. 10.1093/cercor/bhl153 17204823 PMC2689362

[B78] Wang K. Zhang X. Song C. Ma K. Bai M. Zheng R. (2021). Decreased intrinsic neural timescales in mesial temporal lobe epilepsy. *Front. Hum. Neurosci.* 15:772365. 10.3389/fnhum.2021.772365 34955790 PMC8693765

[B79] Xia M. Wang J. He Y. (2013). BrainNet viewer: A network visualization tool for human brain connectomics. *PLoS One* 8:e68910. 10.1371/journal.pone.0068910 23861951 PMC3701683

[B80] Xu Q. Zhang Z. Liao W. Xiang L. Yang F. Wang Z. (2014). Time-shift homotopic connectivity in mesial temporal lobe epilepsy. *AJNR Am. J. Neuroradiol.* 35 1746–1752. 10.3174/ajnr.A3934 24742802 PMC7966270

[B81] Yan C. G. Wang X. D. Zuo X. N. Zang Y. F. D. P. A. B. I. (2016). Data processing & analysis for (resting-state) brain imaging. *Neuroinformatics* 14 339–351. 10.1007/s12021-016-9299-4 27075850

[B82] Yan C. G. Yang Z. Colcombe S. J. Zuo X. N. Milham P. (2017). Concordance among indices of intrinsic brain function:Insights from inter individual variation and temporal dynamics. *Sci. Bull.* 62 1572–1584. 10.1016/j.scib.2017.09.015 36659475

[B83] Yang H. Long X. Y. Yang Y. Yan H. Zhu C. Z. Zhou X. P. (2007). Amplitude of low frequency fluctuation within visual areas revealed by resting-state functional MRI. *Neuroimage* 36 144–152. 10.1016/j.neuroimage.2007.01.054 17434757

[B84] Yang H. Zhang C. Liu C. Yu T. Zhang G. Chen N. (2018). Brain network alteration in patients with temporal lobe epilepsy with cognitive impairment. *Epilepsy Behav.* 81 41–48. 10.1016/j.yebeh.2018.01.024 29475172

[B85] Zang Y. Jiang T. Lu Y. He Y. Tian L. (2004). Regional homogeneity approach to fMRI data analysis. *Neuroimage* 22 394–400. 10.1016/j.neuroimage.2003.12.030 15110032

[B86] Zang Y. F. He Y. Zhu C. Z. Cao Q. J. Sui M. Q. Liang M. (2007). Altered baseline brain activity in children with ADHD revealed by resting-state functional MRI [published correction appears in Brain Dev. 2012 Apr;34(4):336]. *Brain Dev.* 29 83–91. 10.1016/j.braindev.2006.07.002 16919409

[B87] Zeng H. Pizarro R. Nair V. A. La C. Prabhakaran V. (2013). Alterations in regional homogeneity of resting-state brain activity in mesial temporal lobe epilepsy. *Epilepsia* 54 658–666. 10.1111/epi.12066 23294137 PMC4052837

[B88] Zhang Z. Lu G. Zhong Y. Tan Q. Chen H. Liao W. (2010). fMRI study of mesial temporal lobe epilepsy using amplitude of low-frequency fluctuation analysis. *Hum. Brain Mapp.* 31 1851–1861. 10.1002/hbm.20982 20225278 PMC6870704

[B89] Zhang Z. Lu G. Zhong Y. Tan Q. Liao W. Chen Z. (2009). Impaired perceptual networks in temporal lobe epilepsy revealed by resting fMRI. *J. Neurol.* 256 1705–1713. 10.1007/s00415-009-5187-2 19488674

[B90] Zhao B. Yang B. Tan Z. Hu W. Sang L. Zhang C. (2020). Intrinsic brain activity changes in temporal lobe epilepsy patients revealed by regional homogeneity analysis. *Seizure* 81 117–122. 10.1016/j.seizure.2020.07.030 32781401

[B91] Zou Q. H. Zhu C. Z. Yang Y. Zuo X. N. Long X. Y. Cao Q. J. (2008). An improved approach to detection of amplitude of low-frequency fluctuation (ALFF) for resting-state fMRI: Fractional ALFF. *J. Neurosci. Methods* 172 137–141. 10.1016/j.jneumeth.2008.04.012 18501969 PMC3902859

[B92] Zuo X. N. Ehmke R. Mennes M. Imperati D. Castellanos F. X. Sporns O. (2012). Network centrality in the human functional connectome. *Cereb. Cortex* 22 1862–1875. 10.1093/cercor/bhr269 21968567

[B93] Zuo X. N. Kelly C. Di Martino A. Mennes M. Margulies D. S. Bangaru S. (2010). Growing together and growing apart: Regional and sex differences in the lifespan developmental trajectories of functional homotopy. *J. Neurosci.* 30 15034–15043. 10.1523/JNEUROSCI.2612-10.2010 21068309 PMC2997358

